# Metalloproteinases in Endometrial Cancer—Are They Worth Measuring?

**DOI:** 10.3390/ijms222212472

**Published:** 2021-11-19

**Authors:** Kaja Michalczyk, Aneta Cymbaluk-Płoska

**Affiliations:** Department of Gynecological Surgery and Gynecological Oncology of Adults and Adolescents, Pomeranian Medical University, Al. Powstańców Wielkopolskich 72, 70-111 Szczecin, Poland; anetac@data.com

**Keywords:** metalloproteinases, MMP, TIMP, endometrial cancer, cancer

## Abstract

Endometrial cancer is one of the most common gynecological malignancies, yet the molecular mechanisms that lead to tumor development and progression are still not fully established. Matrix metalloproteinases (MMPs) are a group of enzymes that play an important role in carcinogenesis. They are proteases involved in the degradation of the extracellular matrix (ECM) that surrounds the tumor and the affected tissue allows cell detachment from the primary tumor causing local invasion and metastasis formation. Recent investigations demonstrate significantly increased metalloproteinase and metalloproteinase inhibitor levels in patients with endometrial cancer compared to those with normal endometrium. In this review, we aim to show their clinical significance and possible use in the diagnosis and treatment of patients with endometrial cancer. We have critically summarized and reviewed the research on the role of MMPs in endometrial cancer.

## 1. Introduction

Tumor growth, invasion and metastasis are the main characteristic of carcinogenesis and unavoidably lead to cancer-associated death. The degradation of the extracellular matrix (ECM) that surrounds the tumor and the affected tissue allows cell detachment from the primary tumor causing local invasion and metastasis formation [1]. Matrix proteinases (MMPs) are a class of proteinases (similar to serine, aspartatic and cysteine proteinases) that were demonstrated to be able to destroy nearly all components of the extracellular matrix [2,3]. An increased expression and activation of MMPs were found in almost all human cancers [4,5,6]. MMPs were also found to have an impact on various processes that require tissue remodeling including wound healing, ovulation and embryogenesis [2,3]. There has been much research aimed at establishing the role of MMPs in endometrial cancer pathogenesis, yet their exact role remains unknown. In this review, we summarize current knowledge on MMPs and their possible use in the diagnosis, monitoring and treatment of patients with endometrial cancer.

Endometrial cancer is a pathology that is estimated to affect approximately 66,570 patients in 2021 [7]. It is one of the most common gynecologic malignancies, and the number of deaths caused by endometrial cancer is still increasing, yet little is known about the molecular mechanisms that lead to tumor development and progression. As endometrial cancer is often accompanied by early “red flag symptoms” such as uterine bleeding, it is frequently diagnosed in early stages, having good five-year survival rates, with a five-year relative survival of 81.1% [7]. However, even with the use of radical treatment consisting of extensive surgeries, chemo- and radiotherapy, many patients diagnosed at later stages still face early recurrences and/or metastases resistant to conventional therapies.

Endometrial cancer is hormone-dependent and, in particular, an excess of unopposed estrogen was documented to be a major risk factor for the development of endometrial cancer. Historically, endometrial cancer was divided by Bokhman classification into two pathogenetic types: Type I and Type II endometrial cancer [8]. Type I is the more frequent type of EC (endometrial carcinoma) and is usually present in overweight/obese patients with diabetes and is associated with hyperestrogenism and anovulatory uterine bleeding. It is estrogen-associated and has receptor positivity (estrogen/progesterone) and arises due to endometrial hyperplasia. Type II EC is often associated with a poorer prognosis and is more likely to be diagnosed in later clinical stages [9,10]. It arises from atrophic endometrium and is independent of metabolic and endocrine disorders. However, studies have shown that the two endometrial cancer types may share common etiologic factors and that type II EC may not be completely independent [11]. Recent genomic and molecular studies have proposed newer classifications based on specific p53, POLE and PTEN mutations, microsatellite instability and histology [12]. Based on the findings of the Cancer Genome Atlas (TCGA) project, four molecular subtypes of EC were identified: ultramutated—defined by *POLE* mutations, microsatellite instable, copy-number low, and copy-number high tumors [13].

## 2. Metalloproteinases

**Figure 1:**
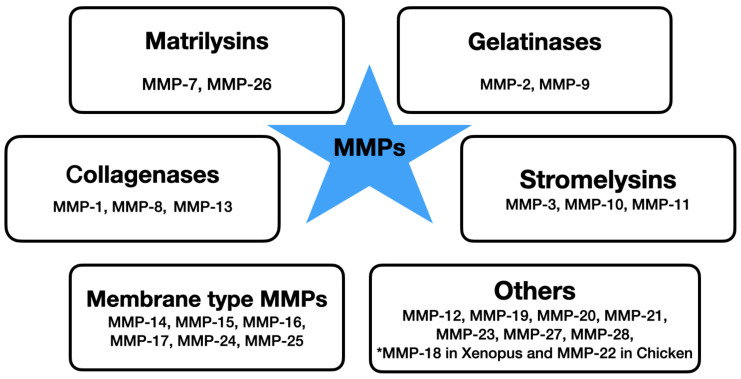
The division of metalloproteinases based on substrate specificity.

Matrix metalloproteinases are a family of 25 metalloproteinases, however only 23 are found in humans as MMP-18 and MMP-22 are not found in Human but in Xenopus and Chicken, respectively. Based on their structure, they are endopeptidases and can be divided into six classes based on their substrate specificity (collagenases, gelatinases, stromelysins, matrilysins, membrane-type MMPs and others) [14] (Figure 1). In accordance with the structural difference, each MMP has the ability to degrade a particular group of matrix proteins. MMPs are zinc-dependent. They are synthesized in the form of inactive zymogens and remain inactive due to the interaction between their prodomain and a zinc ion that is bound to their catalytic site. They can be activated later by the proteinase cleavage. In physiological conditions, their activity is precisely regulated on an mRNA (transcription) level, through activation of precursor zymogens and interaction with specific ECM components. The activity of MMPs can be also regulated by many endogenous inhibitors, including tissue inhibitors of metalloproteinases (TIMPs), molecules with TIMP-like domains and RECK proteins (reversion-inducing cysteine-rich protein with Kazal motifs) [1]. The secretion and activity of metalloproteinases are strictly regulated. Under physiological conditions, MMPs have very low expression and their production is rapidly induced in conditions that require tissue remodeling [15]. Numerous cytokines (i.e., TNF-alpha, IL-1, IL-6), growth factors (TGF-beta, EGF, bFGF), oncogenes, hormones and chemical agents can alter the expression of MMP, either inducing or repressing its levels through important signaling pathways including STAT-3, NF-κB, MAPK and PI3K/Akt pathway [16,17].

MMPs were found to play an important role in tumor invasion, proliferation, infiltration, and metastasis formation [18,19]. Moreover, they were associated with tumor angiogenesis. There is increasing evidence that the expression of MMPs is related to the progression of gynecological malignancies including endometrial cancer [20,21,22,23]. MMP levels can be measured either in serum or tissue specimens. It is debatable if serum concentration of MMPs should be used in research as i.e., MMP-9 was found to be released from cells during serum sampling, increasing its serum concentrations [24]. Moreover, the results can be affected by leucocyte released MMPs during clotting and depending on blood sample collection. In case of MMP measurements, plasma samples should be preferred over serum [25].

## 3. Estrogen-Associated Risk Factors for Endometrial Cancer and Their Effect on MMPs

Estrogens have a mitogenic effect on the endometrium, leading to tissue growth during the menstrual cycle [26]. The estrogen level peaks at ovulation, but the hormones are produced throughout the cycle at lower levels by the corpus luteum before dropping prior to menstruation. Progesterone acts as an inhibitor to the endometrial growth caused by the estrogens. The balance and homeostasis between the two hormone levels are sometimes disrupted by high estrogen levels unopposed of progesterone leading to endometrial hyperplasia and/or cancer [27,28].

MMPs are zinc-dependent proteases participating in the degradation of extracellular matrix proteins including collagen and elastin. During the menstrual cycle, they have a role in the degradation and restoration of the endometrium [29]. Previous research has demonstrated that chronic high levels of estrogen unopposed by progesterone can directly influence and cause the production of MMP-2 and -9 and can also indirectly cause the production of MMP-2 and MMP-9 via the induction of VEGF, interleukin-8, monocyte chemoattractant protein-1 and COX-2 [30,31]. A study by Zhang et al. [32] showed increased expressions of endometrial MMP-2 and -9 and VEGF and the endometrial MVD (microvascular density) in women with anovulatory DUB compared with those in the control group, suggesting that MMPs may play vital roles in the occurrence of irregular uterine bleeding in endometrial hyperplasia of women with anovulatory DUB (dysfunctional uterine bleeding). A study by Shan et al. Further evaluated patients with ADUB and his results showed that the estrogen can increase the expression of VEGF and activate the ERK1/2 pathway to induce MMP-2/9 expression [33]. Also, MMP-26 (endometase) seems to co-variate with the estrogen receptor α (ER-α), as studies have demonstrated high mRNA MMP-26 levels at mid-cycle and patients with endometrial hyperplasia, while sharply declining levels were demonstrated in the second phase of the cycle and in patients with endometrial carcinoma [21,34,35,36]. ER-α was found to be abundant in tissue samples presenting endometrial hyperplasia. ER-α levels decrease gradually with the loss of histopathological differentiation [37,38].

Another important risk factor for EC is obesity, which is closely correlated with excess estrogen levels due to the ability of the adipose tissue to synthesize estrogens [39]. Additional estrogens provide a signal for the endometrial tissue to grow and further proliferate. Obesity is also a risk factor for higher anovulation rates, hormonal imbalance and PCOS (polycystic ovarian syndrome) [40,41,42]. Adipokines produced by the adipose tissue, together with insulin resistance and obesity-induced inflammation can contribute to endometrial cancer development. Matrix metalloproteinases were found to function as modulators of adipogenesis and plasma gelatinase levels were found to be elevated in obese patients [43]. Increased expression of MMPs in obese patients and the role of MMPs in the differentiation of adipocytes may represent a potential molecular link between obesity and cancer [44,45].

## 4. Roles of Metalloproteinases in Carcinogenesis and Tumor Growth

MMPs seem to contribute to tumor growth both at primary and secondary sites. It is probably due to the regulation of the environment through the regulation of access to growth factors from the extracellular matrix that surrounds the tumor either through the exertion of a direct effect or via proteolytic cascade mechanisms [46]. Solid tumors often have a hypoxic environment and to promote their growth create the need for increased transcription factors, growth factors, angiogenic factors and MMPs [47,48]. Molecular changes leading to tumor formation lead to the destruction of intercellular relationships, extracellular matrix (ECM) breakdown and cleavage of basement membrane components by MMP activity modulation, causing disruption of the epithelial-mesenchymal transition (EMT) and promoting cell motility and invasion [5]. EMT relates to the changes that occur in epithelial cells that acquire the characteristics of mesenchymal cells. These transitions occur in physiologic conditions in endometrial tissue as it undergoes remodeling and regeneration during menstruation, decidualization and in preparation for embryo implantation. However, when the MET/EMT (mesenchymal-epithelial transition/epithelial-mesenchymal transition) is altered, the process leads to an increased formation of mesenchymal cells with strong migratory potential and increased invasive properties [49,50]. In vitro studies on endometriosis End1/E6E7 epithelial cells have demonstrated that exogenous addition of active MMP-7 promotes EMT through E-cadherin proteolytic cleavage. Another metalloproteinase, MMP-3 was also reported to induce EMT and increase cellular invasiveness in breast cancer cells [51,52].

MMPs were found to have a role in tumor growth, cell migration and angiogenesis, as they allow the migration of endothelial cells through basal membrane and interstitial ECM for new blood vessel formation [15]. Together with their inhibitors, MMPs seem to regulate sustained tumor growth. The relative MMP levels tend to increase with an increasing tumor stage [46]. Previous research revealed the role of gelatinases in endometrial carcinoma [53,54]. An increasing expression of MMP-2 and decreasing expression of TIMP-2 in endometrial carcinoma tissue correlates with the histological grade of endometrial carcinoma. Moreover, the TIMP-2 expression was found to correlate with the level of myometrial invasion, lymphovascular space involvement as well as lymph node involvement [55]. Similar associations were observed for MMP-9, as it was found to correlate with the histological grade and the disease stage of endometrial carcinoma [56].

## 5. MMPs, Cell Invasion and Metastasis

The metastatic spread of cancer cells remains one of the greatest problems of cancer treatment, therefore it is crucial to understand specific molecular mechanisms responsible for metastasis formation and to create target therapeutic strategies. Metastasis involves a sequence of steps including the escape of cells from the primary tumor site, their entry into the lymphatic and blood circulation system, transport, arrest in distant sites, followed by their growth and the formation of secondary tumors in a new organ environment. Angiogenesis is also one of the crucial points required for the formation of both primary and metastatic tumors and their further growth. MMPs promote the spread of malignant cells through the capillary endothelium and neovascularization [14].

MMPs were demonstrated to be the key regulators of tumor growth both at the primary and metastatic sites and it is believed that they are important for the creation and maintenance of tumor growth [46]. The rationale for the role of MMPs in metastasis originally came from the experiments that manipulated the levels of TIMP-1 that inhibit the function of MMP [46]. Studies using synthetic MMPs provided further evidence for the requirement for MMP activity in the initiation of metastatic foci. MMP inhibitors were shown to reduce the formation of metastasis in melanoma, breast, colon and lung cancer [57,58,59,60].

The effect of MMP activity in metastasis formation has been associated with their ability to degrade the basement membrane and the components of the extracellular matrix, facilitating the invasion of cancer cells through the connective tissue and blood vessels [46]. During the EMT, the features of epithelial cells are lost, favoring the adoption of mesenchymal traits through the loss of apicobasal cell polarity and alternations of intracellular adhesion. This allows the cells to become more motile and invasive, leading to infiltration and the enhanced ability of these cells to migrate [61,62]. MMP-1 was found to be involved in tumorigenesis and metastatic processes through its role as a protease activator receptor 1 (PAR1) agonist [63]. Also, the MMP-9 and MT1-MMP expression in endometrial carcinoma tissue was demonstrated to be significantly associated with the presence of myometrial invasion and vascular/lymphatic invasion [54].

## 6. The Impact of MMPs on Cancer-Associated Angiogenesis

**Figure 2:**
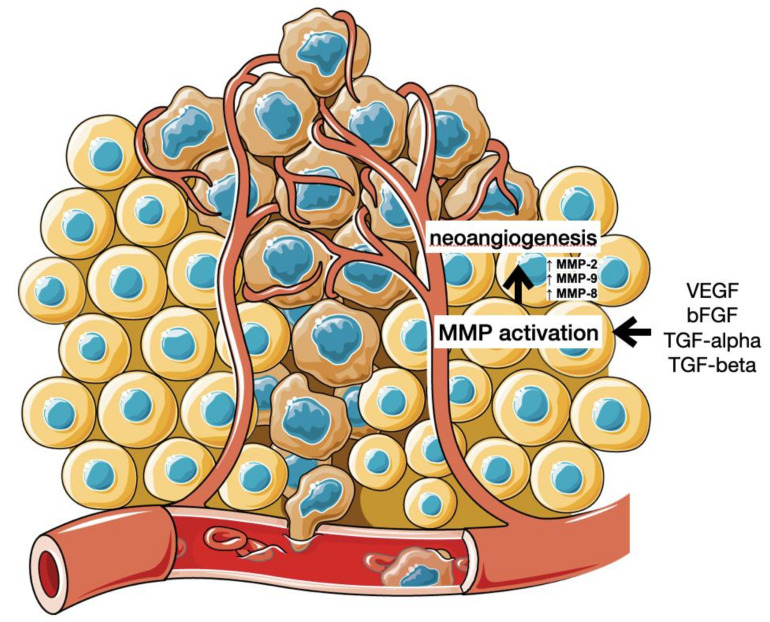
Angiogenesis and cancer metastasis. (Cytokines and growth factors secreted by the cancer cells stimulate endothelial cells to produce and activate MMPs, disrupting ECM and allowing the formation of new blood vessels.).

Angiogenesis is a process of neoformation of blood vessels necessary for cell and tissue metabolism and oxygenation. It has an important and complex role in the human endometrium changes caused by the menstruation cycle and possible blastocyst implantation. Vascular endothelial growth factors (VEGF) play a key role in angiogenesis and its induction in both physiological and pathological processes including malignancy formation. Their activity can be enhanced by various VEGF co-receptors and signaling pathways, while its loss causes the interruption of vasculature development [5]. During angiogenesis, the ECM is degraded by MMPs that facilitate endothelial invasion and lead to the formation of new vessels [1]. The activation of MMP can be induced by angiogenic factors such as VEGF, bFGF (basic fibroblastic growth factors), TGF-alpha and TGF-beta, and angiogenin [5] (see Figure 2).

Normally, angiogenesis is initiated only in the case of inflammation or hypoxia to allow the healing process of tissues and their regeneration. However, it also occurs under pathological conditions such as malignancy processes, as the formation of vascular network is crucial for carcinogenesis as it allows for the propagation and spread of cancer cells resulting in an unfavorable prognosis for the patient [64].

Different MMPs were demonstrated to have different functions and impacts on angiogenesis. An in vitro reduction of cell proliferation and neocapillary network growth and in vivo poor angiogenesis was discovered in animal studies using MMP-8 and MMP-2 knockout mice, demonstrating an evidence for the role of MMPs in angiogenesis processes [65,66]. MMP-9, MMP-14 and MMP-2 have been found to directly regulate angiogenesis, and MMP-9 has been documented to be a key mechanism of the “angiogenic switch” in cancer progression [67,68,69,70]. In human studies, the loss of MMP-1, MMP-8 and MMP-13 were found to cause an irreversible rupture of the extracellular matrix. MMP-1 was shown to promote the expression of VEGFR2 (endothelial growth factor receptor 2) stimulating PAR-1 (serine/threonine-protein kinase) and activating NF-kB transcription factor [71]. Moreover, the cytokines TNF-alpha and IL-8, which have pro-angiogenic properties, were found to stimulate the production of MMPs including MMP-2, MMP-8, and MMP-9. Moreover, MMP-14 was found to significantly contribute to angiogenesis regulation by ECM molecule cleavage and its role as a matrix-degradation enzyme. It is also known as a key effector in the production of VEGF [72]. MMPs and their role in angiogenesis is regulated by TIMPs, which inhibit neovascularization [73].

## 7. MMPs as Diagnostic and Prognostic Markers in EC Patients

**Figure 3:**
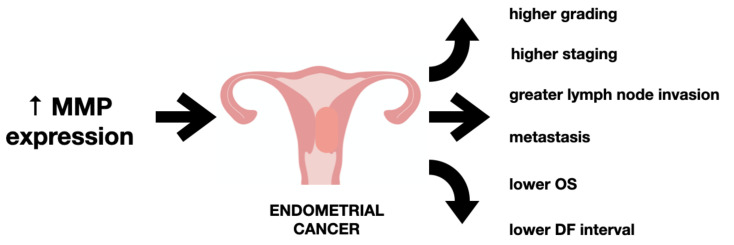
The impact of increased metalloproteinase level on endometrial cancer.

Matrix metalloproteinases facilitate the invasion and destruction of the extracellular matrix as well as further proliferation once the metastasis is formed; this is necessary for cancer spread and metastasis. Myometrial invasion of cancer cells and their further invasion to the local lymph nodes are key features associated with poor prognosis [74] (see Figure 3). Given the physiologic and pathologic roles of MMPs accounting for the changes in the endometrium, MMP expression has been associated with endometrial cancer.

A study by Schröpfer et al. [20] investigated the expression pattern of 23 MMPs in gynecological cancer cell lines. The researchers found MMP-2 expression in Ishikawa cell lines. Moreover, the expression of MMP-11, -23, -24 and -28 was identified in endometrial cancer cell lines both on mRNA and protein level and could potentially be related to the development of endometrial carcinoma. Other studies have also demonstrated MMP-2, -7 and -9 expression in uterine serous and endometrioid carcinoma [75].

The expression of MMPs was found to be related with the progression of gynecological cancers including endometrial carcinoma. Studies have shown an association of MMP-2 and MMP-9 with histopathological grading. Upregulated levels of MMPs was found in patients with higher grading. A similar association was found with the depth of myometrial invasion [54,76]. MMP-2 and TIMP-2 expression were found to be useful as potential markers for endometrial cancer. High MMP-2 and low TIMP-2 expression were correlated with a high risk of both local and distant metastasis [77]. Higher concentrations of MMP2 and MMP-9 were also found in patients with a vascular and/or lymphatic invasion. However, there is still inconclusive data on the levels of MMP, as some studies demonstrate no difference in serum MMP-2 and MMP-9 concentration in endometrial cancer patients, or any associations with the clinical staging of the disease nor with a histopathological grade of the tumor or with age (Honkavouri et al. [78]).

There are limited data concerning survival analysis of MMPs in patients with endometrial carcinoma. A meta-analysis study showed MMP-2 to be closely associated with clinical stage, tumor invasion and metastasis indicating that overexpression of MMP-2 may serve as a poor prognostic factor for patients with endometrial cancer [23]. With regard to MMP-9, a study by Inoue et al. [56] found no correlation between disease outcome and MMP-9 levels. A different study on MMP-7 discovered a correlation between increased expression of MMP-7 in endometrial cancer tissue and greater risk of metastasis [79], higher lymph node invasion [55] and a lower DF (disease-free) interval [80]. A summary of studies on MMP levels in patients with endometrial cancer is listed below in Table 1.

## 8. Metalloproteinase Inhibitors and Future Therapeutic Perspectives in Endometrial Cancer

The activity of matrix metalloproteinases is further modulated through their interactions with their natural inhibitors, which are TIMPs (TIMP-1, TIMP-2, TIMP-3 and TIMP-4). The C-terminal end of TIMP affects the specificity, while the N-terminal allows the formation of complexes with the zinc-bonding site of the active forms of MMPs [84]. They form high-affinity noncovalent complexes with active MMPs, thus inhibiting their action [74]. All TIMPs, except for TIMP-3 which is ECM bound, are present in most tissues and body fluids in a soluble form. The expression of TIMP-1 and TIMP-3, similarly to MMPs, is regulated by growth factors and cytokine levels. Apart from the inhibitory role, TIMPs also have growth factor-like and anti-angiogenic functions [85]. TIMPs, together with MMPs, influence the tumor microenvironment and reorganize tissue structures allowing tumor survival, and spread through the regulation of matrix alignment, structure and immune cell influx. Moreover, in the absence of TIMPs, TIMP-null stromal fibroblasts were found to demonstrate tumor-promoting abilities and to behave like cancer-associated fibroblasts (CAFs) [86]. TIMPs not only exert changes on the local tumor microenvironment but influence the pericellular communication by regulating the bioavailability of protease-dependent growth factor or cytokine signals by inhibiting ADAM proteins [86]. The balance between the amount of active MMPs and free remaining TIMPs determines the net MMP activity [87].

Upregulated TIMP expression may be found in tumor tissue in response to increased MMP levels caused by cancer formation and progression [74]. Overexpression of all TIMPs was found in cancer cell lines of diverse origins and was shown to inhibit cell migration, invasion, metastasis and growth [88]. Increased TIMP-1 levels were found in more advanced-stage tumors as well as endometrial cancer patients with shorter relapse times [78]. Also, high TIMP-2 expression was found to correlate with poor survival of patients suffering from endometrial carcinoma [88].

MMPs and their TIMP inhibitors seem to be promising therapeutic targets for cancer treatment. The correlation between MMP activity and tumor angiogenesis has led to numerous drug development programs, as the tumor growth is limited without the ability of blood supply formation [89]. The use of antiangiogenic therapeutic agents would allow the prevention of new vasculature formation and the normalization of tumor-associated neoangiogenesis [72,90]. The antiangiogenic agents that are already clinically used typically target VEGF and their receptors, or are multikinase inhibitors [90]. Using MMP inhibition as angiogenesis targeting could be an efficacious method. Moreover, this kind of angiogenesis inhibiting therapy could be used in addition to standard chemotherapy to prolong patients’ progression-free and overall survival. However, of concern are potential side effects that may be caused by the use of antiangiogenic therapy including altered healthy tissue blood flow and adverse events such as bleeding, thromboembolism, proteinuria, or hypertension. Their use may also increase drug resistance [90,91,92]. A possible way is the use of monoclonal antibodies to inhibit the activity of MMPs in cancer patients [93]. Moreover, small molecule MMP-9 and MT1-MMP inhibitors seem promising for use in the future due to their novel mechanism of angiogenesis inhibition [89]. Currently, MMP inhibitors are used in clinical trials in the forms of monoclonal antibodies in i.a. breast, gastric, pancreatic, non-small cell lung, esophageal and colorectal cancers [94]. However, to the extent of our knowledge, as of 28 October 2021, no clinical trials of MMP inhibitors in endometrial cancer have been conducted nor registered in the U.S. National Library of Medicine.

The implication of MMPs in tumor invasion and metastasis has prompted the development of strategies that could allow MMP inhibition. TIMPs, as the endogenous inhibitors, attracted considerable attention and promoted research towards the development of the next generation more-specific MMP inhibitors with alternative side groups [86]. Alternations and changes in TIMP structures that directly interact with the MMP substrate-binding site including the AB loop have shown the success of TIMP engineering for specialized MMP inhibition [95,96]. Future alternatives to MMP inhibitors include specific small molecule inhibitors or monoclonal antibodies that are potentially able to target domains other than MMP active sites [97]. Compared to monoclonal antibodies, which are macromolecules, small molecule inhibitors have the advantages of smaller size, better pharmacokinetic properties, better patient compliance and lower costs. Yet, their design and production have to face multiple challenges related to low response rates and cancer drug resistance [98].

The timing and treatment application of MMP inhibitors is critical. Presurgical treatment with oral MMP inhibitor in a mouse breast cancer model improved their survival from 67 to 92% [99]. Moreover, the use of a broad-spectrum MMP inhibitor (marimostat) combined with gemcitabine in a group of patients with pancreatic cancer has significantly improved the survival of patients with disease confined to the pancreas [100]. The creation and application of selective inhibitors could allow the reduction of side effects and avoidance of off-target toxicities.

MMPs participate in multiple processes that regulate carcinogenesis, tumor growth and metastasis formation. An understanding of the molecular mechanisms and roles of MMPs are essential to allow early cancer diagnosis, detection of metastasis formation and production of novel targeted cancer treatments.

## 9. Conclusions

Metalloproteinases seem to have an important role in the pathogenesis and progression of endometrial cancer, yet their exact role in carcinogenesis remains unknown. MMPs, together with their inhibitors, have a role in the regulation of important metabolic and cellular pathways, therefore changes in their homeostasis may lead to carcinogenesis. Multiple factors, including hormonal status and patients’ weight, were found to influence MMP levels. With the evolving use of target treatment, it is important to continue the research on the role and potential use of MMPs in the diagnosis, prognosis and treatment of endometrial cancer.

## Figures and Tables

**Table 1:** The summary of MMP studies on endometrial cancer.

Subgroup	Author	Results	Number of Patients	Level of Evidence *
Collagenases (MMP-1, MMP-8, MMP-13)	Moser et al. [81]	MMP-1 is not associated with OS	103	4
Gelatinases (MMP-2, MMP-9)	Aglund et al. [56]	MMP-2 and MMP-9 correlate with histopathological grade of EC. MMP-9 correlates with clinical staging.	88	4
	Di Nezza et al. [54]	MMP-2, MMP-9 and MT1-MMP (MMP14) correlate with clinicopathological features of EC such as myometrial invasion, vascular/lymphatic invasion and increasing histologic grade	29	4
	Cymbaluk-Płoska et al. [82]	Sighificantly higher serum MMP-9 levels in patients with high-stage, poorly differentiated tumors, blood vessel invasion, lymph node involvement and greater myometrial infiltration.	143	3b
	Yu et al. [83]	MMP-9 levels positively correlated with lymph node metastasis and histopathological grade of EC patients. Higher MMP-9 expression in EC tissue did not correlate with clinical outcome.	188	3b
	Liu et al. [23]	Systematic review and meta-analysis; MMP-2 expression significantly higher in patients with normal endometrium compared to endometriosis/ normal endometria. MMP-2 expression significantly associated with FIGO stage, histologic grade, lymph node metastasis and myometrial invasion.	1902	3a
Stromelysins (MMP-3, MMP-10, MMP-11)	Obokata et al. [81]	MMP-11 may be correlated with the remodelling of normal endometrial lesions; does not correlate with EC	180	3b
Matrilysins (MMP-7, MMP-26)	Obokata et al. [81]	MMP-7 expression in endometrial carcinoma was correlated with myometrial invasion and estrogen receptor expression. The expression of MMP-7 in the adjacent stroma was associated with a poor prognosis.	180	3b
	Misugi et al. [80]	The expression of MMP-7 was significantly stronger in higher-grade than lower-grade tumors. The disease-free interval was significantly shorter when MMP-7 expression was intense. This increased expression of MMP-7 in high grade UECs may be associated with tumor invasion and metastasis, and MMP-7 could serve as a prognostic maker in UEC.	196	3b
	Tunuguntla et al. [21]	Increased MMP-26 staining intensity correlated with grade III tumors and the depth of myometrial invasion	136	3b

* Level of evidence according to Oxford Centre for Evidence-based Medicine (2009). Abbreviations: EC—endometrial carcinoma, OS—overall survival, FIGO—Fédération Internationale de Gynécologie et d’Obstétrique, UEC—uterine endometrial carcinoma.
